# Social Network Analysis of Secure Text Messaging Metadata During Clinical Deterioration in an Inpatient Children’s Hospital Setting

**DOI:** 10.1007/s10916-025-02250-8

**Published:** 2025-09-19

**Authors:** Andrew Harold Smith, Brant Tudor, Vishnu Mohan, Mohamed A. Rehman, Luis Ahumada

**Affiliations:** 1 Division of Critical Care Medicine, Department of Pediatrics, Vanderbilt University School of Medicine, Nashville, TN USA; 2 Department of Biomedical Informatics, Vanderbilt University School of Medicine, Nashville, TN USA; 3 Center for Pediatric Data Science and Analytic Methodology, Johns Hopkins All Children’s Hospital, St. Petersburg, FL USA; 4 Department of Anesthesia and Pain Medicine, Johns Hopkins All Children’s Hospital, St. Petersburg, FL USA; 5 Department of Medical Informatics and Clinical Epidemiology, Oregon Health and Science University, Portland, OR USA; 6 Armstrong Institute, Johns Hopkins Health System, Baltimore, MD USA

**Keywords:** Social network analysis, Information dissemination, Clinical deterioration, Text messaging, Communication

## Abstract

Mitigating clinical deterioration relies upon recognition (afferent limb) and interventions (efferent limb) by a healthcare team. Healthcare provider (HP) communication by text messaging plays a role in facilitating both limbs in the inpatient setting. We sought to quantitatively characterize healthcare provider team communications through the social network analysis (SNA) of secure text messages exchanged in the inpatient setting, and as they relate to a subgroup of patients demonstrating a deterioration during their hospitalization. Messages linked to inpatients exchanged between HPs over a 12-month period, including a cohort of messages linked to patients experiencing deterioration were analyzed using SNA. Subnetworks corresponding to individual patient encounters were constructed, including a series of subnetworks pertaining to patients with an impending clinical deterioration. Network and network participant characteristics were calculated and analyzed. From October 2022 through September 2023 there were 1,065,225 messages delivered by 3,272 HPs, associated with 4,328 inpatient hospital encounters, of which 120 hospital encounters were associated with a deterioration. SNA demonstrated significantly higher measures of eigenvector centrality among frontline providers (FLP) including advanced practice providers and housestaff, relative to attending physician (*p* < 0.001) and registered nurses (*p* < 0.001), consistent with greater influence of the FLP on information dissemination through the entire network. Within individual subnetworks associated with the care of patients experiencing a clinical deterioration, FLP participants demonstrated greater overall network influence (*p* = 0.032) relative to FLP counterparts in networks not associated with a deterioration, despite comparable numbers of participants and connections. Using SNA, we quantitatively characterized a text messaging network within an inpatient hospital setting, demonstrating the importance of FLPs on information dissemination, a finding demonstrated specifically within subnetworks dedicated to the care of individual deteriorating patients. Understanding characteristics of a dynamic communication network of healthcare providers may prove a valuable target in facilitating communication and in mitigating the risks of deterioration.

**IRB Approval**: Johns Hopkins Medicine IRB (#CIR00419339).

**Clinical trial number**: Not applicable.

**Supplementary Information:**

The online version contains supplementary material available at 10.1007/s10916-025-02250-8.

## Introduction

Preventable harm within hospitals accounts for over 210,000 deaths in the US annually. [1] Even more than twenty years ago, the Institute of Medicine’s “Crossing the Quality Chasm” report cites specifically the role of information technology in redesigning our approach to coordinating and delivering high quality care. [2] Despite this report, communication failure remain the root cause for more than 70% of sentinel events, and accounts for a total of $12 billion in excess costs annually in the United States alone. [3, 4] Attention to acute decline in a patient’s clinical condition is time-sensitive that requires both timely recognition and intervention to avert further deterioration and an adverse patient outcome. Along with recognition of a decline in clinical status, escalation in care also requires a response (efferent) limb calling attention and responding to these changes. Indeed, provider responsiveness to nurse-initiated communication is subject to varying perspectives on both the perceived urgency of a clinical situation, as well as factors specific to the communication medium (synchronous or asynchronous) employed. [5]

While increases in the volume of nursing documentation and free-text comments within the electronic record are described in association with patient deterioration or hospital mortality, less is understood concerning asynchronous communication behaviors surrounding acute changes in clinical status. [6, 7] Though short message service (SMS) text messaging has provided one instrument to facilitate text-based communication between clinicians, in 2016 the Joint Commission advocated for the use of secure, encrypted messaging platforms with date and time annotation, message retention, and the facilitation of closed-loop communication through read receipts. [8–10] The implementation of these messaging platforms has been associated with reductions in communication failures and perceived benefits in reliability of communication. [11–13] Encrypted, EHR-based text messaging also provides an opportunity to understand the nature of text communication through interrogation of metadata, particularly given that different members of the healthcare provider team have different perspectives regarding the effectiveness of their communication in the medical decision-making process. [14] Social network analysis (SNA) is the quantitative description and analysis of network participants and their relationships, employing measures of centrality to describe the relative influence of individual participants within a network. [15] We leveraged SNA of secure, encrypted, and de-identified text messaging metadata between healthcare providers over an internal network to quantitatively describe measures of centrality within a network of healthcare providers. We hypothesized that specific healthcare team member roles demonstrated greater influence in the dissemination of information through a network. Furthermore, we hypothesized that this influence is of greater significance with respect to communication between healthcare providers caring for the clinically deteriorating patient.

## Methods

We conducted a retrospective, observational study of text-messaging behaviors by inpatient healthcare providers in a quaternary care freestanding children’s hospital within the United States. Healthcare providers utilized a secure, asynchronous internal messaging platform (Epic Secure Chat, Verona WI) during the study period. De-identified characteristics of messages sent using this platform by an inpatient healthcare provider that were directly linked to an inpatient encounter from October 2022 through September 2023, with at least one recipient were included in this analysis. Hospital encounters were defined as those associated with an admission to an inpatient acute care or intensive care unit bed during the study period. Messages sent but not linked to an inpatient encounter were excluded from analysis. Advanced practice providers and housestaff (clinical fellow and resident physician trainees) responsible for patient-facing care at the direction of attending physicians were collectively grouped as frontline providers (FLP) for the purposes of this analysis. Attending healthcare providers were defined as physicians in this study, responsible for both independently delivering healthcare and the supervision of healthcare delivery by frontline providers. Messages exchanged during the study period were characterized with respect to send date and time, the roles of both sending and receiving provider(s), number of message recipients, minutes elapsed from sent to first read, and message length (character count). Specific message text content was not analyzed, as the scope of this study focused on de-identified message metadata.

A cohort of hospital encounters associated with a clinical deterioration during this same time window was identified. Clinical deterioration for the purposes of this analysis was defined as the activation of the hospital-wide rapid response or code team. [16] Characteristics of the event including primary indication (e.g. respiratory, seizure, sepsis), activating service (e.g. hospitalist), and time of activation relative to hospital admission were identified. Time and date stamps of messages associated with encounters experiencing a clinical deterioration were grouped relative to the time and date of clinical deterioration to the minute, with the deterioration time noted as Time 0.

Social network analysis (SNA) is a collection of methods employed to measure and characterize relationships between connected people or groups. [15] Analysis of such a network in the healthcare setting may facilitate the identification of relationship patterns between network participants, as well as describe how these relationships may modify the behaviors of other participants, or the system collectively. [17] Social network analysis was performed in three stages; the first stage explored a single network incorporating all messages exchanged between inpatient healthcare providers linked to a hospital encounter during the study period. Each node in this network corresponded to an individual healthcare provider, and was also labeled with respect to role within the healthcare team. Directed (sender to recipient) edges between these nodes were weighted according to message volume between unique source and target pairs. The second stage consisted of the creation of subnetworks, with each subnetwork derived from communications exchanged between healthcare provider linked to a unique hospital encounter. Of these subnetworks associated with at least one clinical deterioration during the hospital encounter (“deterioration subnetworks”), subnetworks were constructed from messages exchanged only prior to the clinical deterioration, enabling comparative analysis of each hospital encounter-specific subnetwork. The third stage of analysis focused exclusively on constructing deterioration subnetworks grouped with respect to both (1) unique hospital encounter and (2) timing of messages exchanged relative to the time of a clinical deterioration. Each of these subnetworks were constructed using messages exchanged in distinct 12-hour increments, starting 36 h prior to clinical deterioration up to the time of deterioration (Time 0).

While social network analysis may be depicted visually with participants (nodes) and their directional connections (edges), quantitative analysis of networks and the participants within also included several objective measures. Network characteristics calculated included density (the proportion of connections that exist relative to the total number of possible connections between nodes), eccentricity (the longest distance between a node and all other nodes in a network, where distance is defined as the shortest path between two nodes), diameter (maximum eccentricity, describing the spread of a network), and radius (minimum eccentricity, representing the most central nodes in a network). [18] The global clustering coefficient was also calculated, quantifying the tendency of network participants to form highly connected subgroups. [19] Measures of nodal importance within a network included betweenness centrality (quantifying how frequently a node connects the shortest distance between other node pairs), closeness centrality (quantifying the average shortest distance to all other nodes within the network, with higher values suggesting greater efficiency in information dissemination), and eigenvector centrality. [19] Eigenvector centrality was of particular interest, in that this measure accounts for a network participant’s influence by accounting not only for its number of connections, but also for the influence of nodes to which the participant of interest is connected. [19] Measures of centrality were normalized to allow for inter-network comparison of network participants. Network visualizations were performed using the OpenOrd layout algorithm. [20] After networks were developed and analyzed, descriptive and comparative analyses focused upon roles more directly engaged in patient care across the entirety of a hospital encounter, including registered nursing, nursing assistants, advanced practice providers (nurse practitioners and physician assistants), housestaff (residents and fellows, who together with advanced practice providers were categorized as frontline providers), attending physicians (referred to as physicians in this study), respiratory therapists, and pharmacists. Categorical variables are described as counts and percent of total, with comparisons by chi square and Fisher’s exact testing where appropriate. Continuous variables are described as median (interquartile range [IQR]) and compared using Mann Whitney U or Kruskal-Wallis testing, with Bonferroni correction for multiple comparisons where appropriate. Visual displays were developed using Gephi (v0.10), while network analyses were conducted using Python (v3.11) and iGraph (v0.11.8) [21, 22] Statistical analysis was performed with R (v4.4.0). Code drafting and debugging were performed with the assistance of artificial intelligence (ChatGPT, version 4o mini; OpenAI). This study was approved and waiver for informed consent was provided by the Johns Hopkins Medicine Institutional Review Board (IRB#CIR00419339).

## Results

**Fig. 1:**
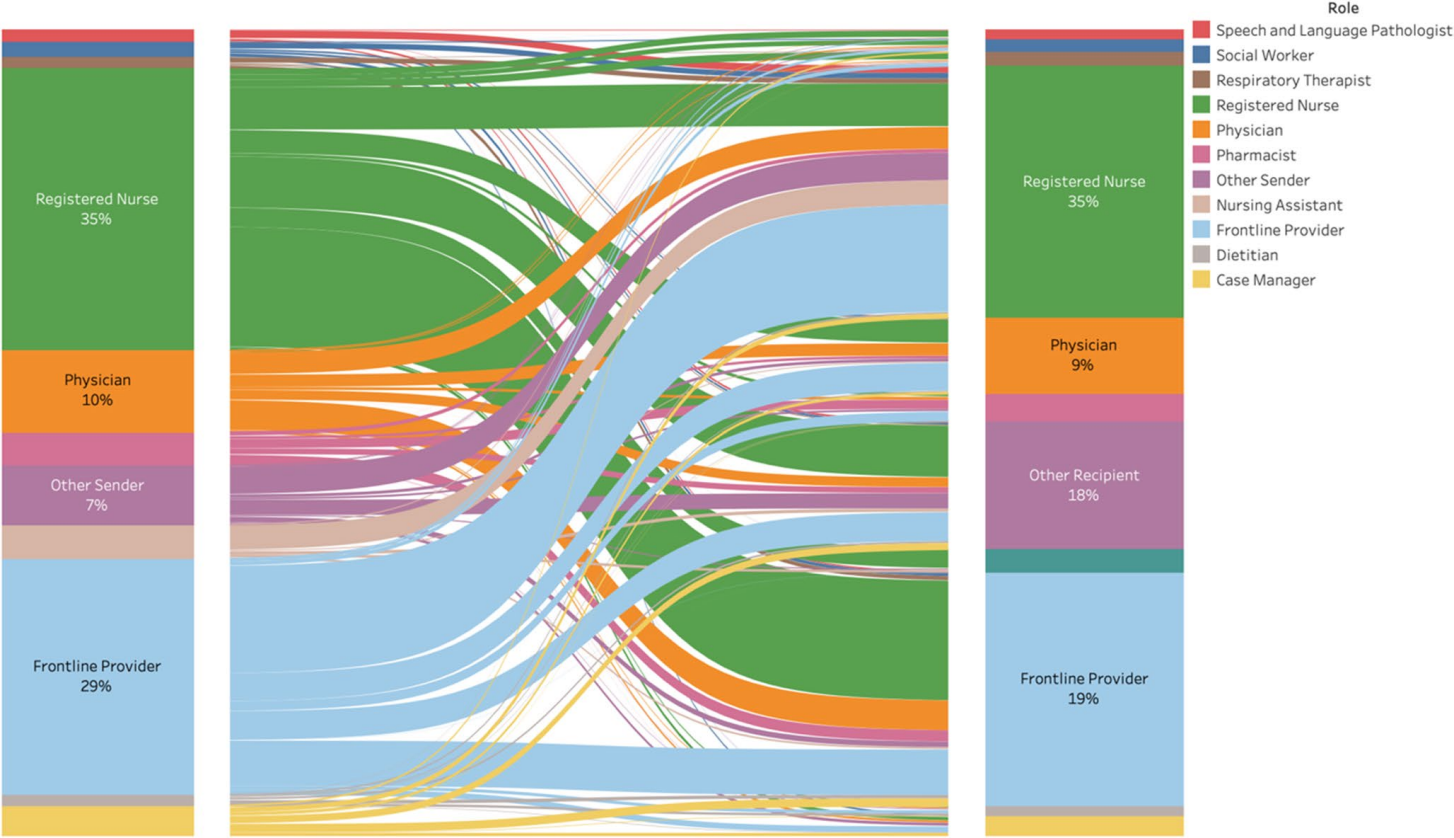
Sankey diagram demonstrating relative distribution of messages by role from outgoing source (left column) to recipient (right column)

From October 2022 through September 2023, there were 1,065,225 messages sent by healthcare team members associated with 3,662 unique inpatients and 4,328 unique inpatient hospital encounters meeting inclusion criteria. Of the 9,305 total inpatient hospital encounters during the study period, secure messaging was directly linked to 47% of these encounters. The majority of messages originated from registered nurses (35%), while advanced practice provider and housestaff roles (collectively referred to as frontline providers) accounted for an additional 29% of message originations. Recipients also were most commonly registered nurse (34%), advanced practice provider (18%) and housestaff (14%) roles (Fig. 1). More than 30 other sender roles categorized as “Other” included roles such as “Non-provider” (*n* = 78) and “Radiology technician” (*n* = 51).

During this same time period, of the 4,328 inpatient encounters there were 120 hospital encounters associated with activation of either a rapid response team or code blue team. Median time to clinical deterioration from admission among those encounters associated with a clinical deterioration was 1.6 days (IQR 0.4–6.6 days), with the majority of deteriorations (*n* = 65, 54%) primarily respiratory in origin. While this population accounted for 2.7% of hospital admissions (*n* = 120 of 4,328 encounters) during the study period within the cohort, they accounted for 8.4% of total hospital days (*n* = 3,568 of 42,369 days) within the cohort, with messages sent related to this cohort accounted for 9.9% of total messaging volume (*n* = 105,648 of 1,065,225 messages).

**Fig. 2:**
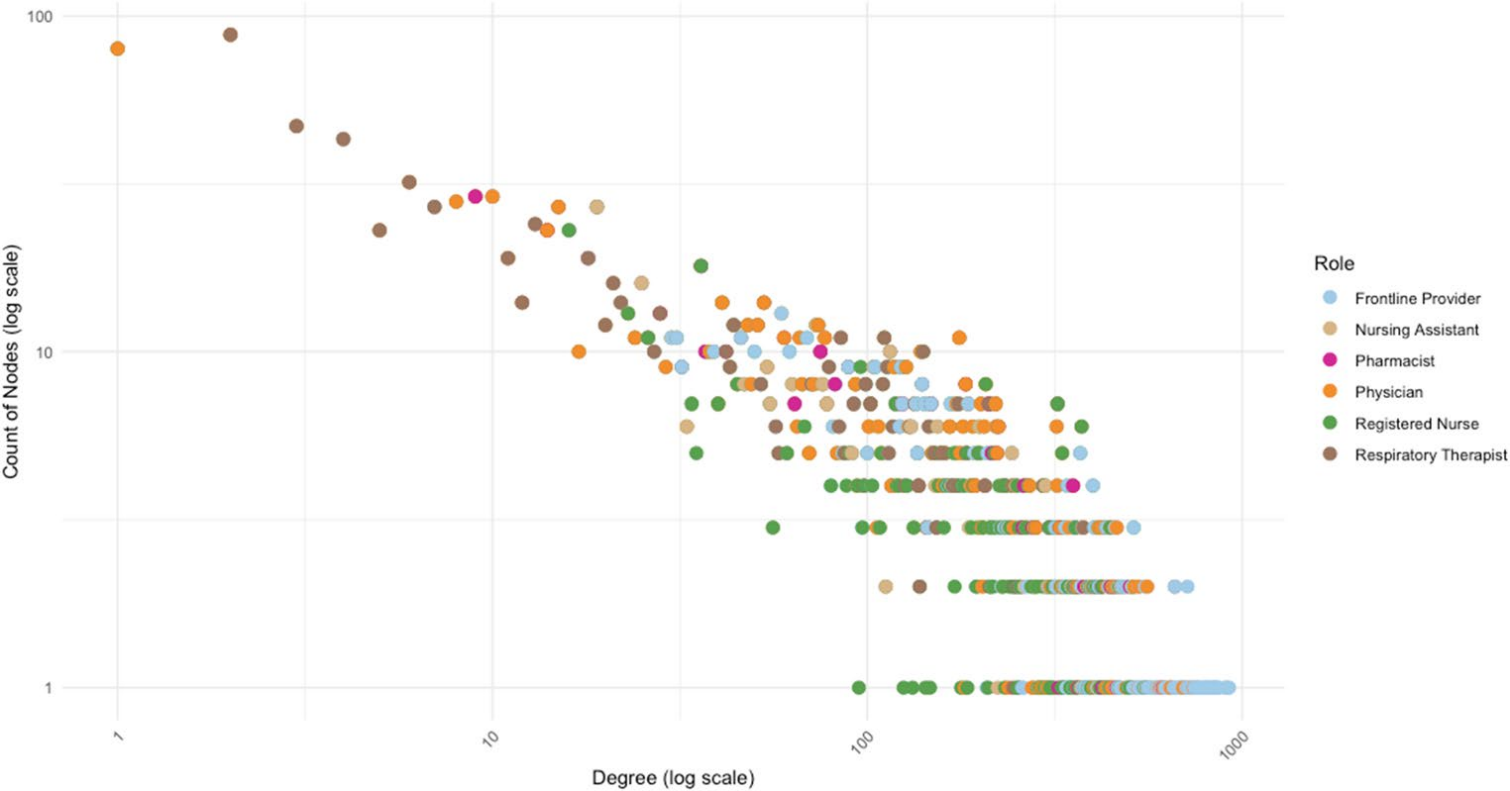
Scaled scatter plotting relating frequency of nodes with number of degrees (incoming and outgoing connections) demonstrating a relatively large proportion of nodes with few degrees, and a smaller proportion of high-degree network participants

**Fig. 3:**
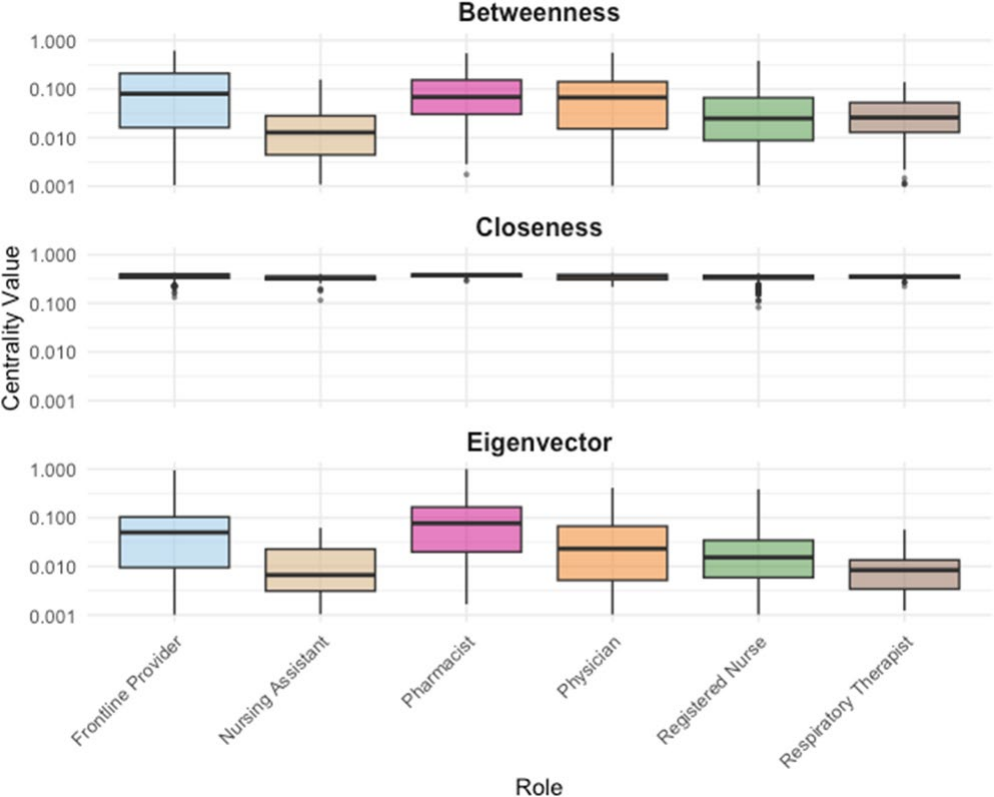
Boxplots summarizing normalized betweenness, closeness, and eigenvector centrality measures among provider roles across the entire network. Boxes represent interquartile range (IQR, 25th – 75th percentile), while horizontal lines within the box represent medians. Whiskers extend to 1.5 x IQR from their respective quartiles, and points beyond whiskers indicate outliers. A logarithmic scale was applied to the Y-axis to accommodate the range of observed values. Frontline providers demonstrated significantly higher eigenvector, closeness, and betweenness centrality relative to both their registered nursing and physician counterparts. Pharmacists also demonstrated significantly higher measures of centrality, including higher eigenvector centrality relative to physician, registered nurse, and frontline providers

A weighted, directed network consisting of directed secure text messages (edges) delivered between healthcare team members (nodes) consisted of 3,272 nodes and 207,753 corresponding edges. Of the 3272 network participants, registered nurses comprised the largest proportion (*n* = 1355, 41%), followed by frontline providers (*n* = 417, 13%) and physician (*n* = 326, 10%) roles. A scaled scatterplot relating frequency of nodes with number of degrees (incoming and outgoing connections) as shown in Fig. 2 demonstrated a relatively large proportion of nodes with few degrees and a small proportion of high-degree network participants. Comparative descriptive features of betweenness, closeness, and eigenvector centrality, with respect to provider role within the network are summarized in Fig. 3 and Supplemental Table 1. Analysis revealed significant variation in these measures across roles most closely involved in patient care, with betweenness, closeness and eigenvector centrality measures all differing by role (*p* < 0.001). Notably, frontline providers demonstrated significantly higher eigenvector and closeness centrality relative to their registered nurse (*p* < 0.001) and physician (*p* < 0.001) counterparts, while betweenness centrality was also significantly higher among frontline providers relative to registered nurses (*p* < 0.001) and physicians (*p* = 0.025). Pharmacists also demonstrated relatively higher measures of centrality, with higher eigenvector centrality relative to physician, registered nurse, and frontline providers (all *p* < 0.001, Supplemental Table 2).

In our analysis of 4,328 hospital encounter-level subnetworks, 106 subnetworks consisted of communications exchanged during a hospital encounter prior to a clinical deterioration. In comparing subnetworks with respect to the presence of a clinical deterioration, there were no significant differences in the numbers of nodes, edges, or network density (Supplemental Table 3). Analysis of centrality measures with respect to role demonstrated significantly higher eigenvector centrality among both frontline providers and nursing assistants within deterioration networks relative to their deterioration-free network counterparts, while closeness centrality tended to be higher across all roles in deterioration-free networks, with the exception of respiratory therapists (Table 1).

**Table 1:** Summary of normalized measures of centrality across 4,314 subnetworks, each corresponding to a unique hospital encounter and stratified by network participant role

Role	Variable	Deterioration network (*n* = 106)*	No deterioration network (*n* = 4208)	*P* value^†^
Frontline Provider	Betweenness centrality	0.00000 (0.00000, 0.01267)	0.00000 (0.00000, 0.01117)	0.52
	Closeness centrality	0.17463 (0.15105, 0.20065)	0.18693 (0.15564, 0.21854)	< 0.001
	Eigenvector centrality	0.00134 (0.00002, 0.00769)	0.00077 (0.00000, 0.00813)	0.032
Nursing Assistant	Betweenness centrality	0.00000 (0.00000, 0.09688)	0.00000 (0.00000, 0.09412)	0.11
	Closeness centrality	0.19008 (0.15172, 0.24111)	0.20000 (0.15385, 0.27048)	0.027
	Eigenvector centrality	0.00324 (0.00000, 0.05772)	0.00209 (0.00000, 0.06010)	0.012
Pharmacist	Betweenness centrality	0.00000 (0.00000, 0.00022)	0.00000 (0.00000, 0.00548)	0.69
	Closeness centrality	0.17438 (0.14213, 0.19187)	0.19183 (0.15736, 0.21704)	0.025
	Eigenvector centrality	0.00010 (0.00000, 0.00154)	0.00047 (0.00000, 0.00585)	0.24
Physician	Betweenness centrality	0.00000 (0.00000, 0.00711)	0.00000 (0.00000, 0.01369)	0.56
	Closeness centrality	0.17779 (0.15644, 0.20275)	0.18815 (0.15599, 0.21652)	0.035
	Eigenvector centrality	0.00094 (0.00000, 0.00675)	0.00107 (0.00000, 0.00950)	0.69
Registered Nurse	Betweenness centrality	0.00000 (0.00000, 0.10709)	0.00000 (0.00000, 0.12209)	0.037
	Closeness centrality	0.19532 (0.15296, 0.24702)	0.20792 (0.15915, 0.28571)	< 0.001
	Eigenvector centrality	0.00363 (0.00000, 0.05502)	0.00300 (0.00000, 0.08168)	0.054
Respiratory Therapist	Betweenness centrality	0.00000 (0.00000, 0.03677)	0.00000 (0.00000, 0.01993)	0.95
	Closeness centrality	0.17563 (0.15038, 0.20833)	0.18747 (0.15413, 0.21932)	0.21
	Eigenvector centrality	0.00145 (0.00002, 0.00715)	0.00130 (0.00000, 0.01047)	0.64

Reported as median (25th, 75th percentile)

*Excludes 14 subnetworks consisting exclusively of communications following deterioration event

^†^Mann-Whitney U test

Analysis of subnetworks in the 36 h prior to clinical deterioration demonstrated significant increases in both nodes (*p* < 0.001) and edges (*p* < 0.001) within a subnetwork as time approached Time 0 (Table 2). While network diameter remained comparable across each epoch, subnetwork clustering coefficients increased significantly in the twelve hour epoch most proximate to Time 0 (Supplemental Table 4). Individual participants within these time-delimited subnetworks demonstrated comparable measures of betweenness (*p* = 0.62) and closeness (*p* = 0.10) centrality over time, along with relative declines in eigenvector centrality (*p* = 0.0014).

**Table 2:** Characteristics of messaging subnetworks and subnetwork participants, with respect to the time of deterioration (Time 0 h)

Variable	Time − 12 to 0 h (*n* = 98)	Time − 24 to −12 h (*n* = 64)	Time − 36 to −24 h (*n* = 51)	*P* value^†^
Betweenness centrality	0.00000 (0.00000, 0.19345)	0.00000 (0.00000, 0.19396)	0.00000 (0.00000, 0.13306)	0.62
Closeness centrality	0.37639 (0.25000, 0.55556)	0.40000 (0.27668, 0.60000)	0.36364 (0.25449, 0.55556)	0.10
Eigenvector centrality	0.05728 (0.00000, 0.54957)	0.09270 (0.00000, 0.67290)	0.20000 (0.00000, 0.68953)	0.0014
Nodes	11.5 (8.0, 19.0)	10.0 (6.0, 17.0)	9.0 (6.0, 15.0)	< 0.001
Edges	19.0 (10.0, 31.0)	15.0 (8.0, 24.0)	13.0 (8.0, 25.0)	< 0.001
Clustering Coefficient	0.19375 (0.00000, 0.32944)	0.17292 (0.00000, 0.27778)	0.18333 (0.00000, 0.35210)	0.0062
Diameter	9.0 (5.0, 13.0)	8.0 (5.0, 13.5)	9.0 (6.0, 11.0)	0.056
Radius	1.0 (1.0, 1.0)	1.0 (1.0, 1.0)	1.0 (1.0, 2.0)	< 0.001

Reported as median (25th, 75th percentile). N describes the number of subnetworks exchanging information and analyzed for a given time epoch

^†^Kruskal-Wallis test

## Discussion

Interdisciplinary communication is instrumental in optimizing care delivery and mitigating the risk of adverse events during the course of a patient’s care. Using social network analysis of a text messaging platform, we characterize text message-based communication relationships between various roles within the healthcare provider team over a 12-month period in an inpatient quaternary care setting. We demonstrate quantitatively that the frontline provider role exerts significant influence over network information flow, as well as a greater degree of influence in conducting the care of patients ultimately experiencing a clinical deterioration during their hospital encounter, relative to frontline providers engaged in the care of patients not experiencing a clinical deterioration. We also characterize networks relative to the timing of a clinical deterioration, noting that while individual participant influence may decrease, there are concomitant increases in network participation in the hours prior to a deterioration.

Timely and effective communication is a vital element to delivering care safely, with communication failures cited by the Joint Commission as a leading root cause of adverse events. [23] The final common pathway to mitigate the trajectory of the clinically deteriorating patient, whether recognized through derangements in physiologic monitoring, alterations in an early warning system, or simple pattern recognition is an escalation in care mediated by an attempt to mobilize resources through broadcasting concerns to available resources. While the use of effective tools including SBAR (situation, background, assessment, recommendation) and critical language have shown promise in facilitating verbal communication between healthcare team members in a highly complex medical environment, relatively less is understood concerning the implications of using text-based means of communication. [3] Indeed it has been posited that with this growth in the use of text message communication come vulnerabilities leading to alert fatigue, inappropriate replacement of critical verbal conversations with text-based communication, and misinterpretation of messaging due to lack of context. [24] One analysis by Hagedorn and colleagues of text messaging patterns between healthcare providers within a children’s hospital outlined a relatively dense and connected central network, comprised of a relatively small number of highly-connected network participants as well as a large number of network participants with relatively few connections. [25] We report here similar findings, but leveraging available message metadata enabled a granular understanding of text communication behaviors between providers related to the care of specific patients over a 12-month time period.

Among measures of centrality examined, eigenvector centrality was of particular interest as this measure accounts not only for the number of connections a node has, but for the connectedness of a node’s connections; therefore while any two nodes may demonstrate a comparable number of connections, a node connected to nodes of greater influence would also relatively higher measures of eigenvector centrality. [19] With higher measures of eigenvector centrality among frontline providers relative to their nursing and physician counterparts, we quantitatively demonstrated that the frontline provider role exerted significantly greater influence upon information flow through the secure text messaging network. Consistent with an inpatient clinical setting, we also demonstrate objectively that the frontline provider role more effectively serves as an intermediary directing the transit of information between other nodes (betweenness centrality) and greater efficiency in the dissemination of information across an entire network (closeness centrality) relative to both nurses and physicians. Interestingly, we also demonstrated relatively higher measures of eigenvector, betweenness, and closeness centrality among pharmacists in this network; indeed the role of the clinical pharmacist in the inpatient setting has been independently associated with significant reductions in resource utilization and mortality, along with improved medication safety practices in the inpatient setting. [26, 27] Their relative importance in this network may suggest an organizational prioritization of this role in the inpatient setting, or potentially the pharmacist’s preferred reliance upon secure text messaging relative to other communication modalities.

In addition to describing overall network behavior, the linkage of message metadata with hospital encounter features enabled the creation of individual, encounter-specific subnetworks. By creating these individual encounter-specific subnetworks, and further classifying these networks as related to the care of a patient ultimately experiencing a clinical deterioration, among frontline providers we demonstrate significantly higher eigenvector centrality values and a greater degree of influence in networks associated with the care of the clinically deteriorating patient, relative to frontline providers caring for patients in deterioration-free subnetworks. While frontline providers in these deterioration networks demonstrate greater connectedness to other influential nodes within a deterioration network, also demonstrated were significantly lower measures of closeness centrality in these same networks. These findings suggest that while frontline providers caring for the deteriorating patient may be members of a tightly-connected and highly influential group within a network dedicated to the care of the deteriorating patient, they also tend to be less centrally-located and further removed from other groups within that same network. Indeed, lower closeness centrality was observed in virtually all other roles caring for the clinically deteriorating patient, a finding potentially explained by greater multidisciplinary communication demands and resultant longer, more indirect communication paths. Betweenness centrality values were also zero for many nodes within the encounter-specific networks. As betweenness centrality quantifies the frequency with which a node lies on the shortest path between two other nodes, a betweenness centrality value of zero is consistent with a node that does not serve as an intermediary in any shortest path. Given the median betweenness centrality was 0 for all roles in both deterioration and deterioration-free networks, these findings suggest that the encounter-specific networks were relatively fragmented, with many nodes existing in isolated pairs or small clusters lacking intermediary connections.

As social networks are dynamic and subject to change with respect to both numbers, influence, and connectedness of participants over time, these changes may impact the ease by which information may traverse a network. Given the timing of messages was a known factor among patients experiencing a clinical deterioration in this study, we were able to quantitatively demonstrate changes in an individual’s subnetwork of healthcare providers as time approached a clinical deterioration. While we demonstrate an increase in both the number of participants (nodes) and the number of connections (edges) within a network as time approached a deterioration, we also demonstrated decreases in clustering coefficient after the first twelve hour epoch that subsequently increased in the twelve hours preceding a deterioration; while these initial decreases in clustering coefficient may represent a joining of disconnected nodes or a tendency toward one-to-many broadcast communications to disconnected recipients, subsequent increases in clustering coefficient approaching Time 0 in turn may reflect more sustained engagement of participants within network subgroups. We also demonstrated concomitant decreases in an individual node’s influence on subnetwork behavior (eigenvector centrality) over the same time window. As eigenvector centrality measures the influence of a node based upon its connections to other influential nodes within the network, decreases in network influence over time may be secondary to participants (appropriately) transitioning to alternative, synchronous forms of communication as a patient’s clinical status declines; future comprehensive studies incorporating the analysis of alternative means of communication between healthcare team members will be an important step in more fully characterizing interdisciplinary communication practices during the care of the clinically deteriorating patient.

This study was subject to several limitations. Text messaging is by no means the sole means of promoting interdisciplinary clinical communication, and in the setting of a clinically deteriorating patient requiring timely interventions, the importance of closed-loop, synchronous communication including telephone-based or “face to face” conversations escalate. Within this series alone, 47% of encounters were linked to text message exchanges; while a proportion of inpatient admissions may not have required interdisciplinary communication beyond face to face or telephone conversations during the course of care, identification of messages related to a clinical deterioration required end users to have actively appended a unique patient identifier to the message(s) thus serving as a barrier to complete capture of the message corpus. Furthermore, as investigators were not able to analyze the work schedules of healthcare team participants, this study design did not permit an understanding of network participation relative to their total time caring for patients in the inpatient setting. While this effort focused upon message metadata, unstructured message content was not analyzed as a part of this investigation; future efforts incorporating message content through natural language processing methods holds promise in identifying any objective relationships between changes in communication behaviors and a patient’s clinical state that can be leveraged to mitigate the risk of deterioration. [28]

## Conclusions

In conclusion, we objectively characterize secure text message communication relationships between various roles within a healthcare provider team over a 12-month period in an inpatient quaternary care setting. We demonstrate quantitatively that the frontline provider role exerts significantly greater network influence relative to registered nurse and attending physician team members, both within an entire network and within a subset of communications related specifically to the care of patients experiencing a clinical deterioration during an inpatient hospital encounter. Future efforts integrating physiologic, assessment and communication tools into a comprehensive measure of clinical status and patient trajectory may serve as a valuable complement to the identification of patients at risk for clinical deterioration to further enhance communication and the safe delivery of healthcare in the inpatient setting.

## Supplementary Information

Below is the link to the electronic supplementary material.

Supplementary Material 1 (DOCX 25.4 KB)

## Data Availability

No datasets were generated or analysed during the current study.

## Declarations

### Protection of Human and Animal Subjects

The study was performed in compliance with the World Medical Association Declaration of Helsinki on Ethical Principles for Medical Research Involving Human Subjects. This study was approved and waiver for informed consent was provided by the Johns Hopkins Medicine Institutional Review Board (IRB#CIR00419339).
