# Supplementary material for: Social Network Analysis of Secure Text Messaging Metadata During Clinical Deterioration in an Inpatient Children’s Hospital Setting
Source: J Med Syst. 2025 Sep 19;49(1):116. doi: 10.1007/s10916-025-02250-8 (PMC12449403; doi:10.1007/s10916-025-02250-8)
Supplement: Supplementary file 1 — Supplementary Material 1 (DOCX 25.4 KB) [file 10916_2025_2250_MOESM1_ESM.docx]

**Supplemental Table 1.** Summary of normalized betweenness, closeness, and eigenvector centrality measures with respect to provider roles across the entire network.

| **Measure** | **Frontline Provider** | **Nursing Assistant** | **Pharmacist** | **Physician** | **Registered Nurse** | **Respiratory Therapist** | **P value^†^** |
| --- | --- | --- | --- | --- | --- | --- | --- |
| Betweenness centrality | 0.04377  (0.00177, 0.16296) | 0.00447  (0.00048, 0.01986) | 0.0651  (0.02084, 0.14019) | 0.02224  (0.00039, 0.10144) | 0.01402  (0.00149, 0.04781) | 0.01903  (0.00403, 0.04661) | <0.001 |
| Closeness centrality | 0.37211  (0.32818, 0.39861) | 0.33576  (0.30689, 0.36018) | 0.38342  (0.35618, 0.39648) | 0.35484  (0.30755, 0.3901) | 0.34978  (0.31589, 0.37328) | 0.3568  (0.33205, 0.37424) | <0.001 |
| Eigenvector centrality | 0.02611  (0.00345, 0.08877) | 0.00361  (0.00064, 0.0126) | 0.07685  (0.01984, 0.1655) | 0.00642  (0.00065, 0.03952) | 0.00871  (0.00118, 0.02692) | 0.00594  (0.0017, 0.01248) | <0.001 |

Reported as median (25th, 75th percentile).

^†^Kruskal-Wallis test.

**Supplemental Table 2.** Summary of *post hoc* pairwise comparisons of centrality measures with respect to provider role across the entire network.

|  |  |  | **Role 2** | | | | |
| --- | --- | --- | --- | --- | --- | --- | --- |
| **Measure** | **Role 1** |  | Frontline Provider | Nursing Assistant | Pharmacist | Physician | Registered Nurse |
| **Closeness centrality** | Nursing Assistant |  | **<0.001** |  |  |  |  |
|  | Pharmacist |  | 1.0 | **<0.001** |  |  |  |
|  | Physician |  | **0.01** | **<0.001** | **0.008** |  |  |
|  | Registered Nurse |  | **<0.001** | **0.001** | **<0.001** | 0.33 |  |
|  | Respiratory Therapist |  | **0.009** | **<0.001** | **<0.001** | 1.0 | 1.0 |
|  |  |  |  |  |  |  |  |
|  |  |  | Frontline Provider | Nursing Assistant | Pharmacist | Physician | Registered Nurse |
| **Betweenness centrality** | Nursing Assistant |  | **<0.001** |  |  |  |  |
|  | Pharmacist |  | 1.0 | **<0.001** |  |  |  |
|  | Physician |  | **0.025** | **<0.001** | **0.018** |  |  |
|  | Registered Nurse |  | **<0.001** | **<0.001** | **<0.001** | 0.21 |  |
|  | Respiratory Therapist |  | **0.009** | **<0.001** | **<0.001** | 1.0 | 1.0 |
|  |  |  |  |  |  |  |  |
|  |  |  | Frontline Provider | Nursing Assistant | Pharmacist | Physician | Registered Nurse |
| **Eigenvector centrality** | Nursing Assistant |  | **<0.001** |  |  |  |  |
|  | Pharmacist |  | **<0.001** | **<0.001** |  |  |  |
|  | Physician |  | **<0.001** | 0.089 | **<0.001** |  |  |
|  | Registered Nurse |  | **<0.001** | **<0.001** | **<0.001** | 1.0 |  |
|  | Respiratory Therapist |  | **<0.001** | 1.0 | **<0.001** | 1.0 | 0.16 |

*Post hoc* pairwise comparisons performed with Mann-Whitney U test with Bonferroni correction.

**Supplemental Table 3.** Summary of network characteristics across 4,314 subnetworks, each corresponding to a unique hospital encounter associated with a clinical deterioration event during the hospital encounter.

| **Variable** | **Deterioration networks** (n=106)* | **No deterioration networks** (n=4208) | **P value^†^** |
| --- | --- | --- | --- |
|  |  |  |  |
| Nodes | 24.5  (8.25, 51.5) | 25.0  (14.0, 44.0) | 0.30 |
| Edges | 42.5  (12.5, 116.25) | 45.0  (23.0, 89.0) | 0.42 |
| Clustering Coefficient | 0.196  (0.100, 0.252) | 0.166  (0.086, 0.239) | 0.097 |
| Diameter | 16.0  (7.0, 27.0) | 17.0  (10.0, 24.0) | 0.45 |
| Radius | 1.0  (1.0, 1.0) | 1.0  (1.0, 1.0) | 0.55 |
| Density | 0.203 (0.095, 0.364) | 0.171 (0.105, 0.288) | 0.14 |

Reported as median (25^th^, 75^th^ percentile).

*Excludes 14 subnetworks consisting exclusively of communications following deterioration event.

^†^Mann-Whitney U test

**Supplemental Table 4.** Summary of *post hoc* pairwise comparisons of centrality measures and network properties with respect to time of deterioration (Time 0).

|  | Time 1 | Time 2 | |
| --- | --- | --- | --- |
| Betweenness centrality |  | -12 to 0 hrs | -24 to -12 hrs |
|  | -24 to -12 hrs | 1.0 |  |
|  | -36 to -24 hrs | 0.98 | 1.0 |
| Closeness centrality |  |  |  |
|  | -24 to -12 hrs | 0.13 |  |
|  | -36 to -24 hrs | 1.0 | 0.31 |
| Eigenvector centrality |  |  |  |
|  | -24 to -12 hrs | 1.0 |  |
|  | -36 to -24 hrs | **0.0012** | **0.012** |
| Node count |  |  |  |
|  | -24 to -12 hrs | **<0.001** |  |
|  | -36 to -24 hrs | **<0.001** | 0.14 |
| Edge count |  |  |  |
|  | -24 to -12 hrs | **<0.001** |  |
|  | -36 to -24 hrs | **<0.001** | 1.0 |
| Clustering coefficient |  |  |  |
|  | -24 to -12 hrs | **0.011** |  |
|  | -36 to -24 hrs | 1.0 | **0.026** |
| Diameter |  |  |  |
|  | -24 to -12 hrs | 0.054 |  |
|  | -36 to -24 hrs | 0.77 | 0.99 |
| Radius |  |  |  |
|  | -24 to -12 hrs | **0.024** |  |
|  | -36 to -24 hrs | 0.37 | **<0.001** |

*Post hoc* pairwise comparisons performed with Mann-Whitney U test with Bonferroni correction.
